# Total Joint Arthroplasty in Ehlers–Danlos Syndrome Is Associated with Increased Instability and Revision: A Systematic Review and Meta-analysis

**DOI:** 10.1007/s43465-026-01752-y

**Published:** 2026-03-08

**Authors:** Ibrahim M Muhammad, Maha F Valiyakathu, Mustafa Yousaf, Yusuf Hussain, Chris Bretherton

**Affiliations:** 1 Barts and The London School of Medicine and Dentistry, Queen Mary University of London, 4 Newark Street, London, E1 2AT UK; 2 Blizard Institute, Bone and Joint Health, Queen Mary University of London, 4 Newark Street, London, E1 2AT UK

**Keywords:** Ehlers–Danlos syndrome, Total joint arthroplasty, Hip arthroplasty, Knee arthroplasty, Joint instability

## Abstract

**Background:**

Total joint arthroplasty (TJA) has been increasingly performed in younger and medically complex patients. Ehlers–Danlos syndrome (EDS), a heritable connective tissue disorder characterised by ligamentous laxity and soft tissue fragility, may predispose patients to adverse arthroplasty outcomes, yet existing evidence remains fragmented.

**Methods:**

A systematic review and meta-analysis was conducted in accordance with PRISMA guidelines. Comparative studies evaluating postoperative outcomes following primary TJA in patients with EDS versus non-EDS controls were identified from MEDLINE, Embase, Web of Science, and CENTRAL through November 2025. Random-effects meta-analyses were performed to estimate pooled risk ratios (RRs) for revision, instability or dislocation, aseptic loosening, periprosthetic fracture (PPFx), periprosthetic joint infection (PJI), wound complications, medical complications, and readmission or reoperation.

**Results:**

Nine retrospective cohort studies encompassing over 415,000 patients were included. Compared with non-EDS patients, those with EDS demonstrated a significantly higher risk of instability or dislocation (RR 2.93, 95% CI 2.37–3.61) and all-cause revision (RR 1.97, 95% CI 1.29–3.02), particularly following total hip arthroplasty. Aseptic loosening and wound complications occurred more frequently in EDS patients, though associations were less consistent across models. No significant differences were observed in risks of PJI, medical complications, PPFx, or readmission or reoperation.

**Conclusions:**

Patients with EDS undergoing TJA face substantially increased risks of mechanical complications, notably instability and revision, while infection and medical complication risks are comparable to those of non-EDS patients. These findings highlight the importance of targeted preoperative counselling and surgical strategies aimed at optimising joint stability in this high-risk population.

## Introduction

Total joint arthroplasty (TJA) is among the most effective surgical interventions for end-stage joint disease, providing substantial improvements in pain, function, and health-related quality of life. Procedures such as total hip arthroplasty (THA) and total knee arthroplasty (TKA) are performed with increasing frequency worldwide, driven by population ageing, expanding indications, and advances in implant design, surgical technique, and perioperative care [1]. As utilisation grows, TJA is increasingly offered to younger patients and those with complex medical or musculoskeletal conditions, underscoring the importance of accurately defining procedure-related risks in specific patient populations [2, 3].

Although outcomes following TJA are generally excellent, complications including instability or dislocation, aseptic loosening, PPFx, infection, wound complications, medical morbidity, and revision surgery remain clinically relevant [4]. A range of patient-related factors has been associated with adverse postoperative outcomes; however, evidence regarding outcomes in patients with rare heritable connective tissue disorders remains limited [5–7].

Ehlers–Danlos syndromes (EDS) represent a heterogeneous group of inherited connective tissue disorders characterised by abnormalities in collagen synthesis or processing. Common clinical features include generalised joint hypermobility, ligamentous laxity, soft tissue fragility, impaired wound healing, and chronic musculoskeletal pain [8]. While EDS has traditionally been considered rare, emerging data suggest that it may be underdiagnosed [9, 10]. From an orthopaedic perspective, these pathophysiological features raise concern regarding joint stability, implant fixation, and soft tissue integrity following arthroplasty, potentially predisposing patients with EDS to higher rates of mechanical complications and revision [11–13].

Several retrospective cohort studies have examined outcomes following primary arthroplasty in patients with EDS, reporting variable findings across different joints and outcome domains [14–16]. However, the existing literature is characterised by small sample sizes, heterogeneous study designs, variable follow-up durations, and inconsistent reporting of complications. Consequently, the overall risk profile of patients with EDS undergoing TJA remains incompletely defined.

The aim of this systematic review and meta-analysis was to synthesise the available comparative evidence on postoperative outcomes following primary total joint arthroplasty in patients with Ehlers–Danlos syndrome compared with non-EDS patients. Specifically, we evaluated risks of revision surgery, instability or dislocation, aseptic loosening, PPFx, periprosthetic joint infection (PJI), wound complications, medical complications, and readmission or reoperation across arthroplasty procedures.

## Methods

### Systematic Review Protocol

This systematic review was conducted in accordance with the Preferred Reporting Items for Systematic Reviews and Meta-Analyses (PRISMA) 2020 guidelines [17]. The study protocol was registered with the International Prospective Register of Systematic Reviews (PROSPERO Registration Number: CRD420261284091).

### Study Eligibility Criteria

Studies were selected according to the Population, Exposure, Comparator, Outcomes, and Study Design (PECOS) framework [18]. The inclusion criteria comprised comparative studies published in English that included patients undergoing TJA with one cohort consisting of patients diagnosed with EDS and a comparator cohort of non-EDS patients. Eligible studies were required to report at least one predefined outcome of interest, including revision, instability or dislocation, aseptic loosening, PPFx, PJI, wound complications (surgical site infection, wound complications, cellulitis, or haematoma), medical complications (pneumonia, cardiac events, urinary tract infection, acute kidney injury, deep vein thrombosis, pulmonary embolism, or sepsis), or readmission or reoperation. Instability/dislocation was defined as a composite outcome encompassing frank dislocation, subluxation, ligamentous laxity, and implant-related instability; in THA, this predominantly reflected prosthetic dislocation events identified through diagnostic coding, whereas in TKA it included broader clinical instability (e.g. ligamentous or insert-related), and these were grouped due to inconsistent distinction across administrative and institutional studies.

Exclusion criteria included non-comparative studies, case reports, case series, conference abstracts, review articles, cadaveric or biomechanical studies, non-English publications, and studies that did not involve patients undergoing total joint arthroplasty or did not report relevant postoperative outcomes.

### Search Methods for Identification of Studies

A comprehensive search was performed across MEDLINE, Embase, Web of Science, and CENTRAL from inception to 30 November 2025, following consultation with an institutional librarian. The search strategy incorporated controlled vocabulary and free-text terms related to arthroplasty and joint replacement, including “arthroplasty,” “joint replacement,” “total joint arthroplasty,” “total joint replacement,” “total hip arthroplasty,” “total hip replacement,” “THA,” “total knee arthroplasty,” “total knee replacement,” “TKA,” “shoulder arthroplasty,” “elbow arthroplasty,” and “ankle arthroplasty,” in combination with terms related to Ehlers–Danlos syndrome and connective tissue disorders, including “Ehlers–Danlos syndrome,” “EDS,” and “connective tissue disorder.

### Study Screening, Data Extraction, and Risk of Bias

Two independent reviewers (MY and YH) screened titles and abstracts for eligibility, followed by full-text assessment of potentially relevant articles. Discrepancies during screening or data extraction were resolved through discussion with a third reviewer (IM).

Data were extracted using a standardised data collection form. Where required information was not available, corresponding authors of the respective studies were contacted to obtain additional data. If outcome data were not reported in a study and no response was received from the corresponding authors, the outcome was considered unavailable and excluded from the analysis.

As all included studies evaluating outcomes in patients with Ehlers–Danlos syndrome were non-randomised, methodological quality and risk of bias were assessed using the Risk Of Bias In Non-randomised Studies of Interventions (ROBINS-I) tool [19]. ROBINS-I was selected as it is specifically designed to assess risk of bias in non-randomised comparative intervention studies. Two reviewers (MY and MFV) independently performed the assessment, with disagreements resolved by consensus or consultation with a third reviewer (IM).

### Statistical Analyses

Statistical analyses were conducted with EDS status as the primary exposure. The intervention group comprised patients with EDS, and the comparator group comprised patients without EDS. Outcomes were analysed across studies reporting postoperative complications following TJA. Where studies reported outcomes separately for THA and TKA, these data were retained and incorporated as predefined subgroups.

For each outcome, absolute event risks for the EDS and non-EDS groups were summarised descriptively as crude pooled proportions, calculated as the total number of events divided by the total number of patients across all included studies for each group. Exact 95% confidence intervals (CIs) for these proportions were calculated using the binomial method to provide absolute risk context alongside relative effect estimates.

Comparative meta-analyses were performed using the Mantel–Haenszel (M-H) method to calculate risk ratios (RRs) with corresponding 95% CIs. A random-effects model was specified a priori as the primary analytical approach to account for anticipated clinical and methodological heterogeneity between studies, including differences in patient populations, surgical procedures, perioperative management, and follow-up duration. The Hartung–Knapp adjustment was applied to derive more conservative confidence intervals for the pooled random-effects estimates. In addition, a fixed-effect model was calculated and presented alongside the random-effects model as a sensitivity analysis to assess the robustness of results to modelling assumptions. For outcomes with rare events, specifically wound complications and periprosthetic fracture, fixed-effect Mantel–Haenszel models with treatment arm continuity correction were used to provide more stable effect estimates.

Subgroup analyses were conducted according to surgical procedure (TKA vs THA) using random-effects models, with subgroup-specific pooled RRs, 95% CIs, p-values, and heterogeneity estimates reported. Differences between subgroups were assessed using the χ^2^ test for subgroup interaction as implemented in the meta-analytic framework.

Statistical heterogeneity was quantified using the I^2^ statistic, with values of approximately 0–25, 25–50, and > 50% interpreted as low, moderate, and substantial heterogeneity, respectively. The number of studies contributing to each outcome was recorded and reported.

To explore potential small-study effects, funnel plots were constructed for outcomes with more than five contributing studies and assessed visually for asymmetry. No formal statistical tests for funnel plot asymmetry were performed due to limited power and the presence of sparse data. All statistical tests were two-sided, with *p*-values ≤ 0.05 considered statistically significant. 95% CIs are reported throughout.

## Results

**Fig. 1:**
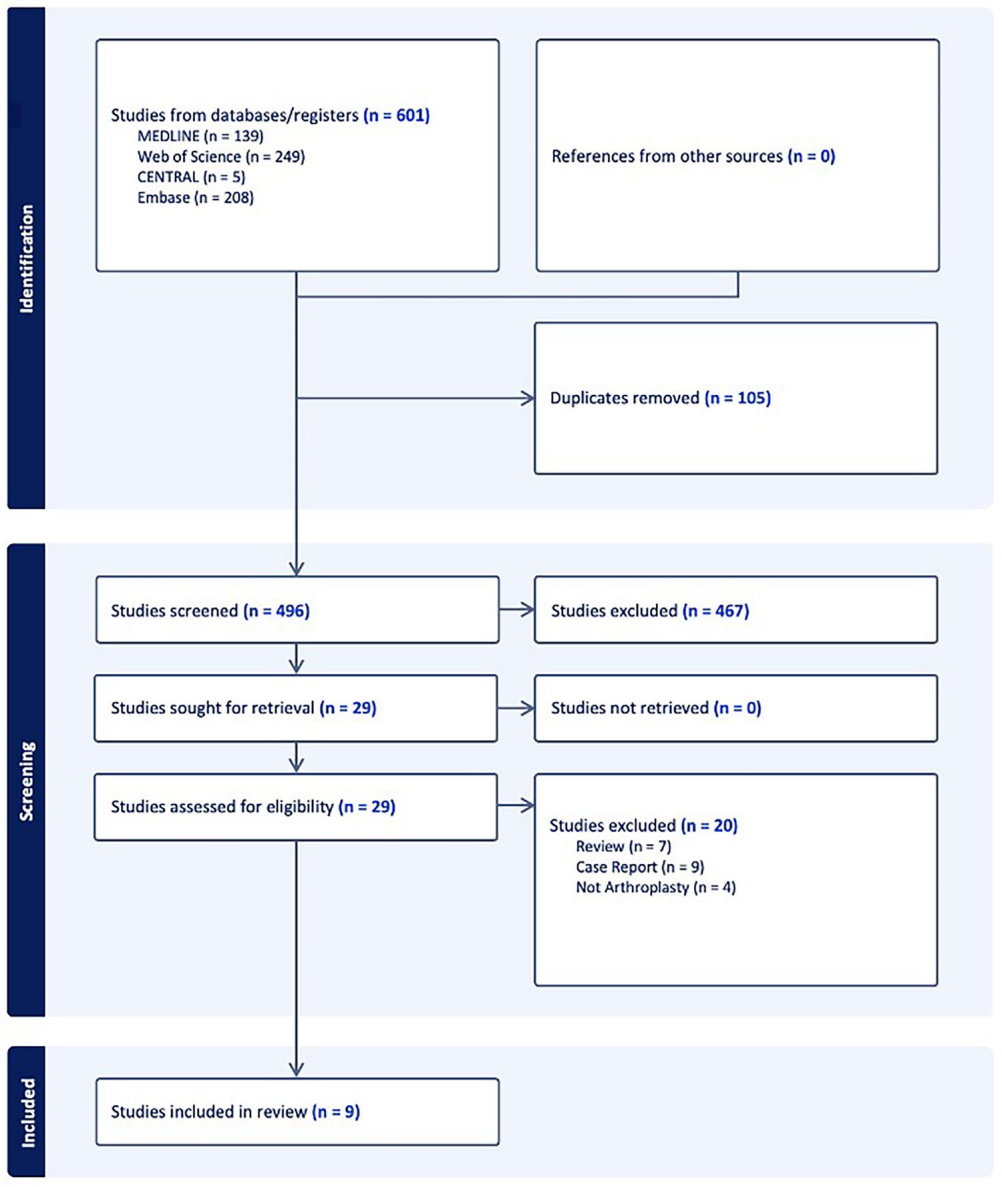
PRISMA flow diagram showing study selection process for inclusion in the systematic review

The initial search yielded 601 records, with 496 remaining after duplicate removal. Subsequently, titles and abstracts were screened according to the predefined eligibility criteria, resulting in the exclusion of 467 records. Full-text assessment was then conducted for the remaining 29 studies, of which 9 studies met the inclusion criteria and were included in the final review (Fig. 1).

### Study Characteristics

The nine included studies were retrospective cohort studies (level IV evidence) published between 2019 and 2025, including three studies on TKA, five studies on THA and one study on total shoulder arthroplasty (TSA). Study periods spanned from 1997 to 2021. Sample sizes varied substantially, ranging from small, matched cohorts of fewer than 50 participants to large database studies including over 400,000 patients. Across studies, patients with Ehlers–Danlos syndrome (EDS) were matched to non-EDS controls using demographic and comorbidity variables such as age, sex, comorbidity indices, and relevant medical conditions. Mean or median patient age was generally in the fifth to sixth decade of life, and the proportion of female participants was high across all studies. Minimum follow-up periods ranged from 3 months to 2 years, where reported. Study characteristics and demographic data are summarised in Table 1 and outcomes reported by each study in Table 2 (Appendix).

**Table 1:** Characteristics of included studies evaluating arthroplasty outcomes in patients with EDS and matched non-EDS controls

Study, year, country	Study design	Level of evidence	Study years	Procedure	Matching variables	Mean/median age EDS	Mean/median age non-EDS	Female % EDS	Female % non-EDS	Minimum follow-up	Sample size EDS	Sample Size non-EDS	MINORS Score /24
Fuqua, 2024, USA [14]	Retrospective cohort	IV	2009–2020	TKA	Matching variables	57	58	77	76	6 months	188	752	16
Fuqua, 2025, USA [15]	Retrospective cohort	IV	2009–2020	THA	Age, sex, chronic renal, pulmonary, hepatic, and cardiovascular disease; prior venous thromboembolism; malignancy; Charlson comorbidity index; mood disorders	56	55	85	88	2 years	118	418	16
Guier, 2020, USA [12]	Retrospective cohort	IV	1997–2017	THA	Age, sex, chronic renal, pulmonary, and hepatic disease; cardiovascular disease; prior venous thromboembolism; malignancy; Charlson comorbidity index; mood disorders	53.8	53.8	76.9	76.9	2 years	13	39	16
Kelly, 2024, USA [20]	Retrospective cohort	IV	2015–2020	THA	Age, sex, length of follow-up	54.3	50.8	84	59	Not reported	238	405,013	16
Kubsad, 2024, USA [11]	Retrospective cohort	IV	2010–2021	TKA	Age, sex, obesity	55–64 median	55–64 median	85.7	85.8	2 years	540	2153	16
		IV	2010–2021	THA	Age, sex, Charlson comorbidity index	45–54 median	45–54 median	82.9	83.0	2 years	340	1353	16
Moore, 2022, USA [13]	Retrospective cohort	IV	2010–2018	THA	Age, sex, Charlson comorbidity index	56.4	56.5	85.3	85.3	3 months	354	3518	16
Rogers, 2021, USA [16]	Retrospective cohort	IV	2007–2018	TSA	Age, sex, Elixhauser comorbidity index; diabetes; chronic kidney disease; obesity; coronary artery disease; congestive heart failure	55.9 ± 12	57.7 ± 10.5	100	90	25 months	10	20	16
Shichman, 2024, USA [21]	Retrospective cohort	IV	2009–2020	THA	Age, sex, year of surgery; prosthesis type; matched 1:2	51.7	51.7	98	98	1 year	66	660	16
Tibbo, 2019, USA [22]	Retrospective cohort	IV	2001–2016	TKA	Age, sex, race; insurance status; Elixhauser comorbidity index	57	56	81.3	80.0	2 years	20	40	16

### Quality of Studies

**Fig. 2:**
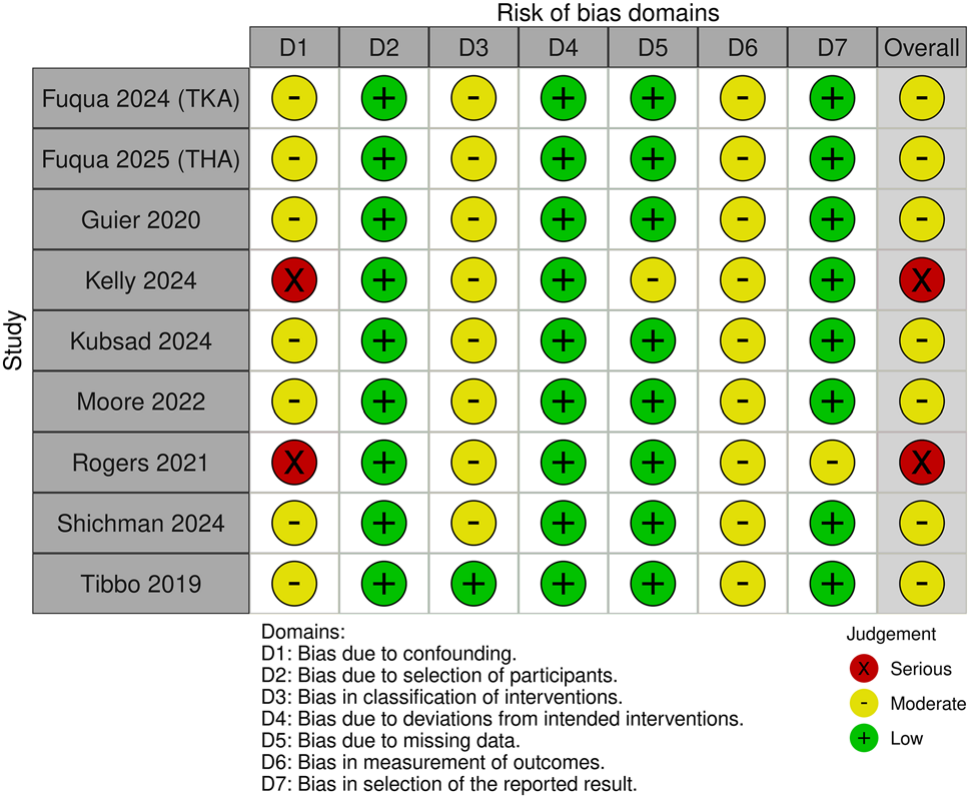
Risk of bias assessment of included studies using the ROBINS-I tool

Based on the ROBINS-I assessment, seven of the nine included studies were judged to have an overall moderate risk of bias, while two studies were rated as having a serious risk of bias (Fig. 2). Risk of bias most commonly arose from confounding (Domain 1) and outcome measurement (Domain 6), which were frequently rated as moderate or serious. All studies were retrospective in design, and outcomes were primarily derived from registry data, administrative coding, or retrospective chart review, increasing the potential for residual confounding and measurement bias. Risk of bias related to participant selection, intervention classification, deviations from intended interventions, and missing data was generally low across studies.

### Risk of Publication Bias

Small-study effects/publication bias were explored using funnel plots where ≥ 5 studies were available; plots are presented in Supplementary Appendix (Figures 7, 8, 9, 10, 11 and 12). Visual inspection did not suggest marked asymmetry, although interpretation is limited by the small number of studies for most outcomes.

### All-Cause Revision

**Fig. 3:**
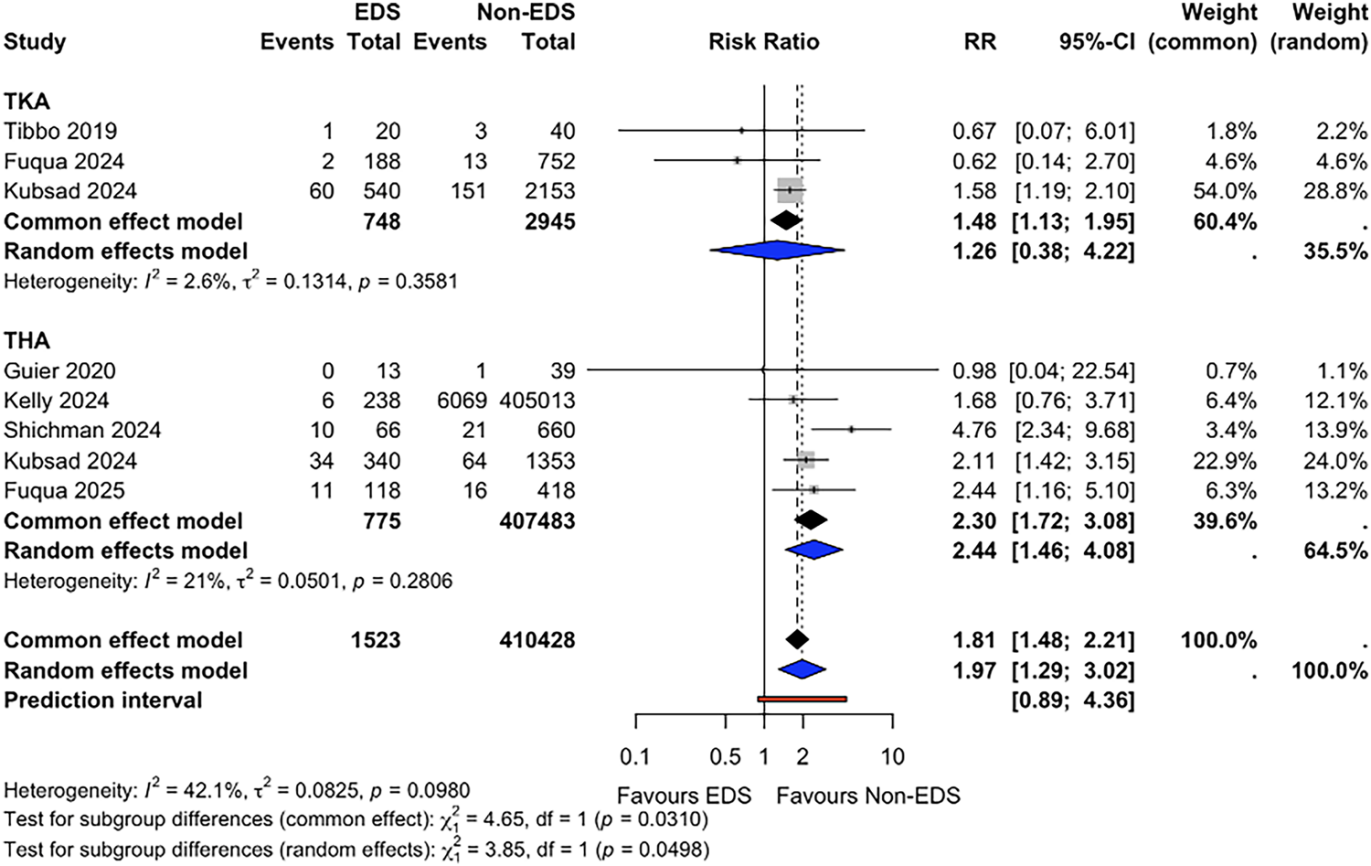
Forest plot comparing all-cause revision risk between patients

All-cause revision risk was reported in eight studies (*n* = 411,951). The pooled all-cause revision risk during the follow-up period was 8.1% (95% CI 6.8–9.6) for patients with EDS and 1.5% (95% CI 1.5–1.6) for non-EDS patients. Meta-analysis suggested a higher risk of all-cause revision in the EDS group (RR 1.97, 95% CI 1.29–3.02, *p* = 0.007; I^2^ = 0.4%) (Fig. 3). Fixed-effect sensitivity analysis demonstrated a statistically significant association (RR 1.81, 95% CI 1.48–2.21, *p* < 0.001). In subgroup analyses, no statistically significant difference was observed in TKA studies (RR 1.26, 95% CI 0.38–4.22, *p* = 0.492; I^2^ = 0.0%), whereas THA studies suggested a higher risk of revision in the EDS group (RR 2.44, 95% CI 1.46–4.08, *p* = 0.009; I^2^ = 0.2%). Fixed-effect subgroup sensitivity analyses demonstrated a statistically significant association in TKA studies (RR 1.48, 95% CI 1.13–1.95, *p* = 0.005) and THA studies (RR 2.30, 95% CI 1.72–3.08, *p* < 0.001). It should be noted that given the short follow-up periods across included studies (range 3 months to 2 years), these revision estimates predominantly capture early mechanical failures and may not reflect long-term implant survivorship.

### Instability or Dislocation

**Fig. 4:**
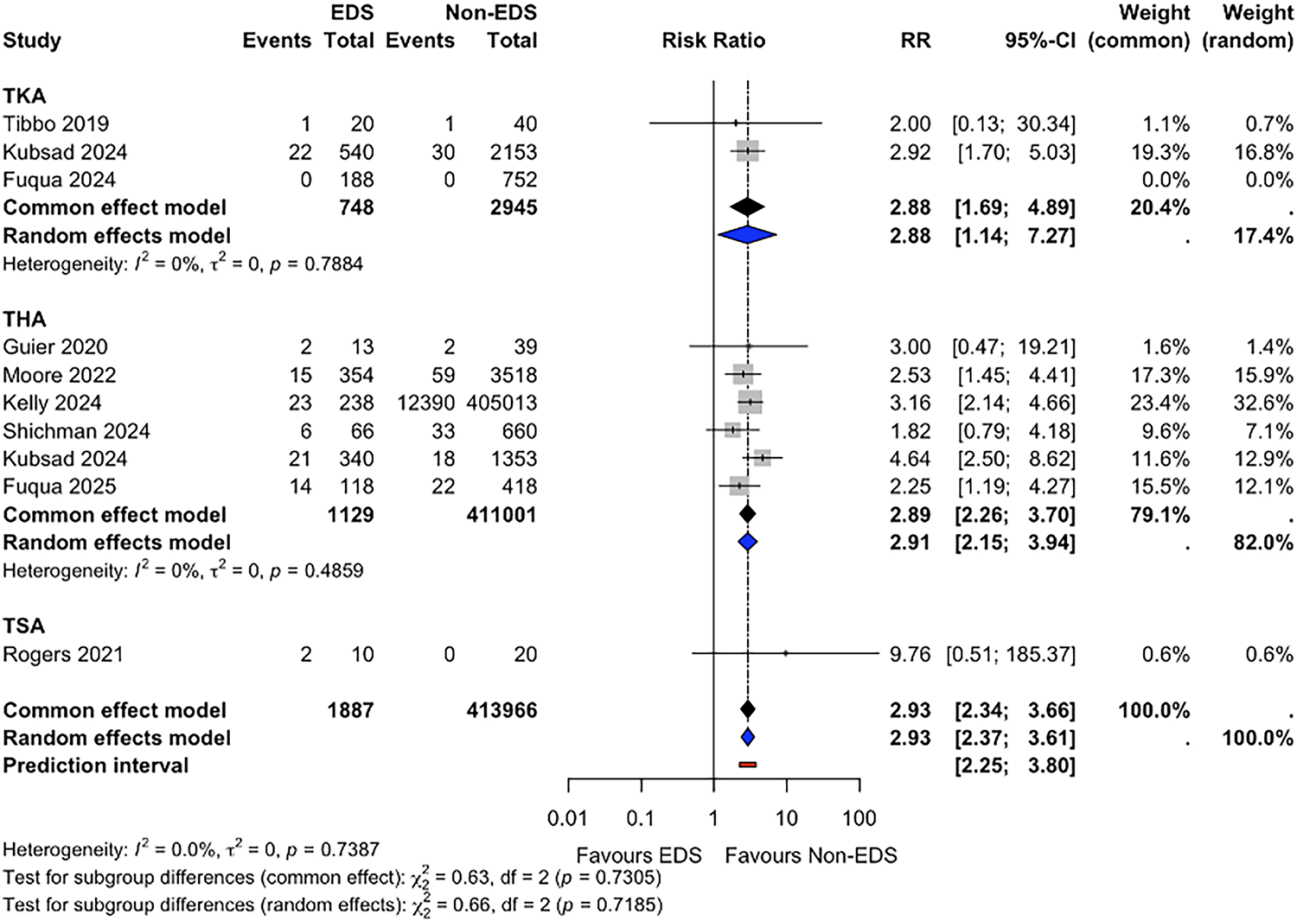
Forest plot comparing instability or dislocation risk between patients

Instability or dislocation risk was reported in 9 studies (*n* = 415,853). The pooled instability or dislocation risk during the follow-up period was 5.6% (95% CI 4.6–6.8) for patients with EDS and 3.0% (95% CI 3.0–3.1) for non-EDS patients. Meta-analysis suggested a higher risk of instability or dislocation in the EDS group (RR 2.93, 95% CI 2.37–3.61, *p* < 0.001; I^2^ = 0.0%) (Fig. 4). Fixed-effect sensitivity analysis yielded consistent results (RR 2.93, 95% CI 2.34–3.66, *p* < 0.001). In subgroup analyses, a statistically significant difference was observed in both TKA studies (RR 2.88, 95% CI 1.14–7.27, *p* = 0.044; I^2^ = 0.0%) and THA studies, suggesting a higher risk of instability or dislocation in the EDS group (RR 2.91, 95% CI 2.15–3.94, *p* < 0.001; I^2^ = 0.0%).

### Aseptic Loosening

**Fig. 5:**
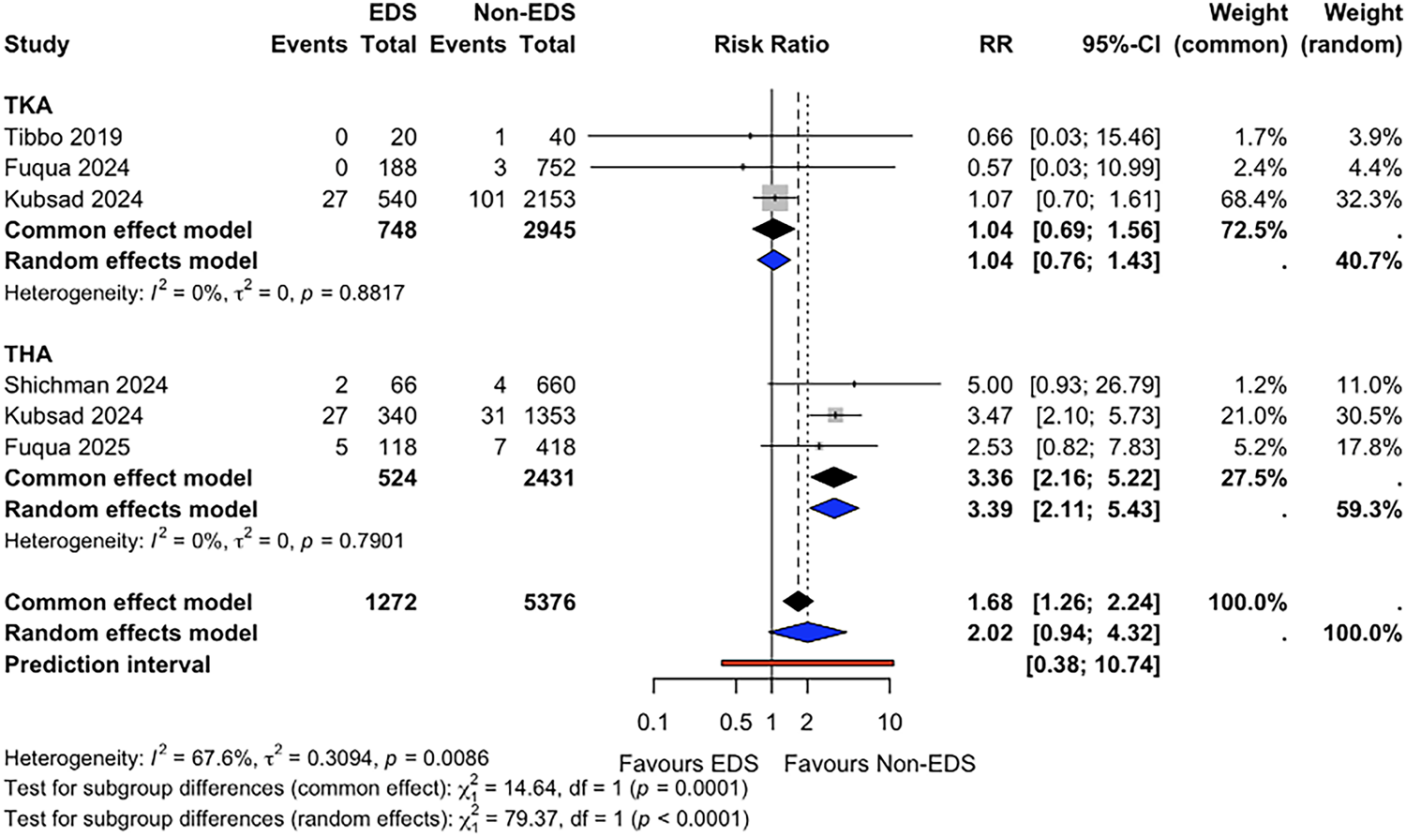
Forest plot comparing aseptic loosening risk between patients

Aseptic loosening risk was reported in 6 studies (*n* = 6648). The pooled aseptic loosening risk during the follow-up period was 4.8% (95% CI 3.7–6.1) for patients with EDS and 2.7% (95% CI 2.3–3.2) for non-EDS patients. Meta-analysis suggested a higher risk of aseptic loosening in the EDS group, although this did not reach statistical significance (RR 2.02, 95% CI 0.94–4.32, *p* = 0.065; I^2^ = 0.7%) (Fig. 5). Fixed-effect sensitivity analysis demonstrated a statistically significant association (RR 1.68, 95% CI 1.26–2.24, *p* < 0.001). In subgroup analyses, no statistically significant difference was observed in TKA studies (RR 1.05, 95% CI 0.76–1.43, *p* = 0.611; I^2^ = 0.0%), whereas THA studies suggested a higher risk of aseptic loosening in the EDS group (RR 3.39, 95% CI 2.11–5.43, *p* = 0.008; I^2^ = 0.0%). Fixed-effect subgroup sensitivity analyses yielded consistent findings.

### Periprosthetic Fracture

**Fig. 6:**
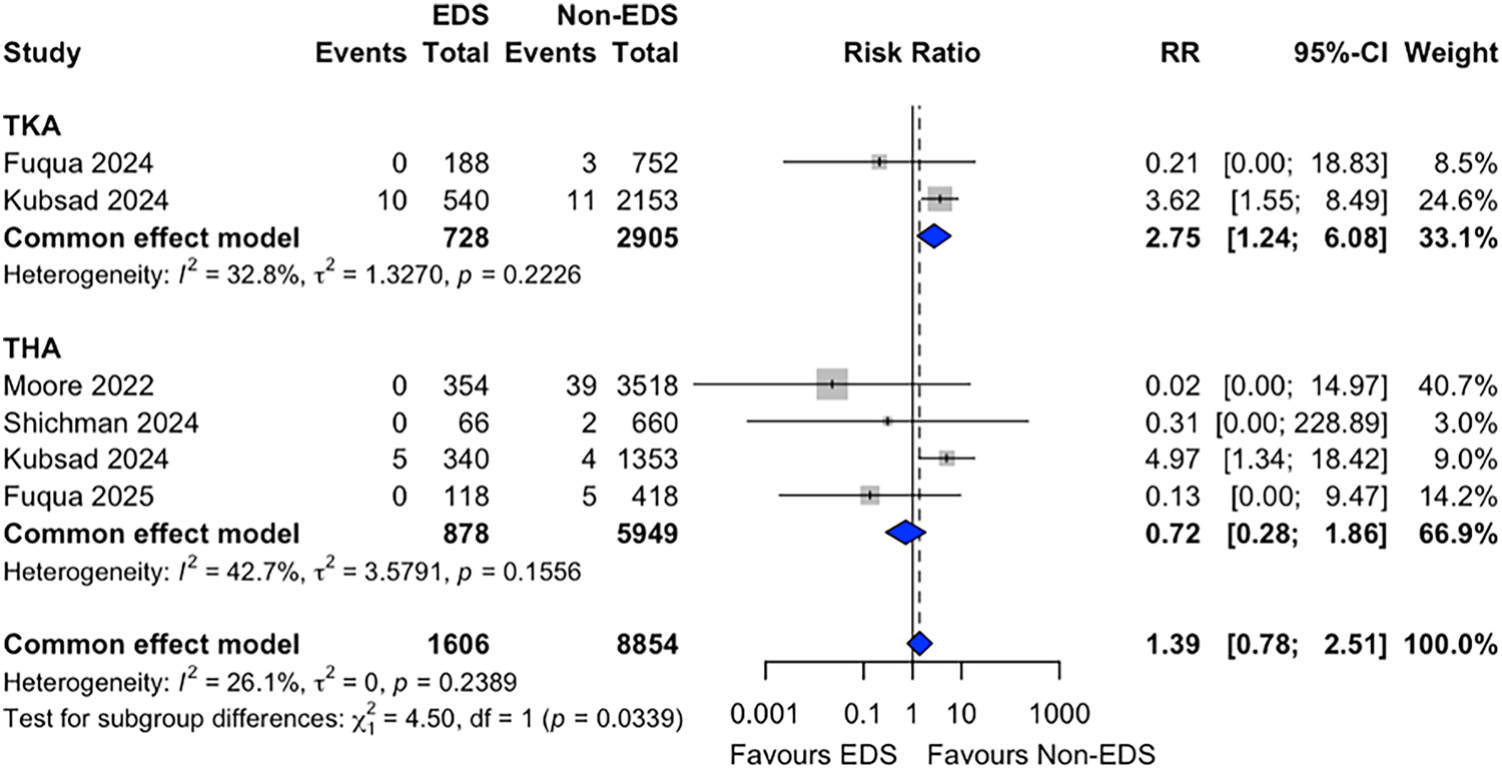
Forest plot comparing periprosthetic fracture risk between patients

**Fig. 7:**
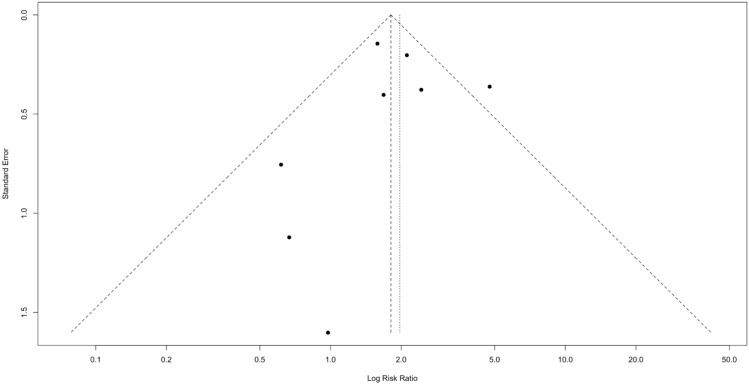
Funnel plot assessing publication bias for all-cause revision outcomes

**Fig. 8:**
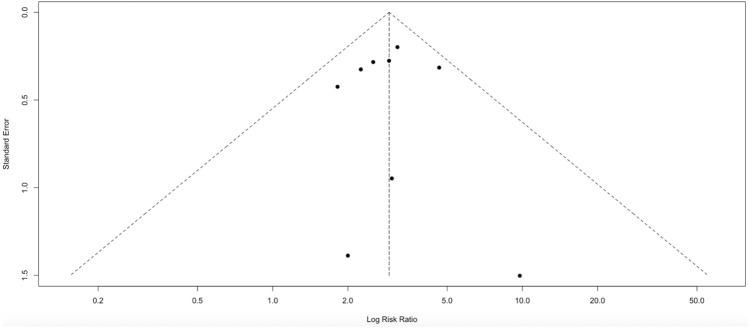
Funnel plot assessing publication bias for instability or dislocation outcomes

**Fig. 9:**
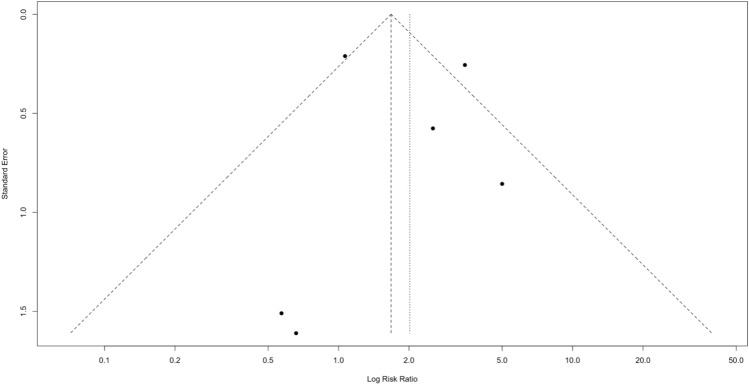
Funnel plot assessing publication bias for aseptic loosening outcomes

**Fig. 10:**
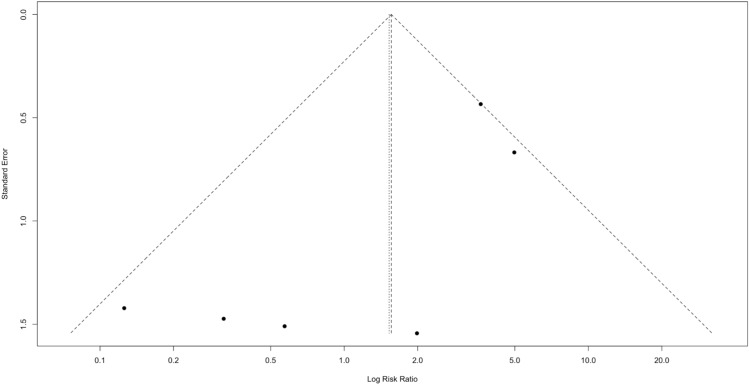
Funnel plot assessing publication bias for periprosthetic fracture outcomes

**Fig. 11:**
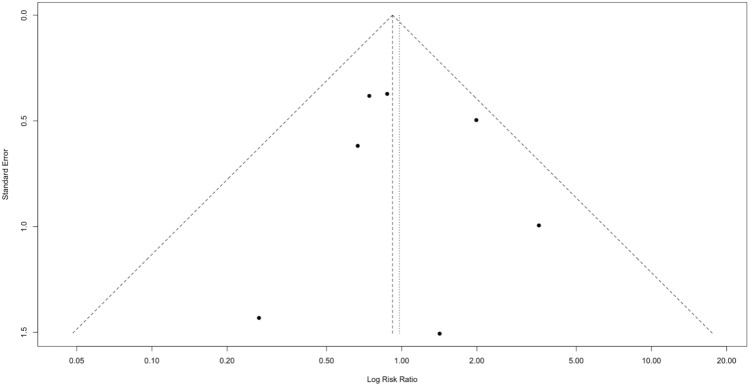
Funnel plot assessing publication bias for periprosthetic joint infection outcomes

**Fig. 12:**
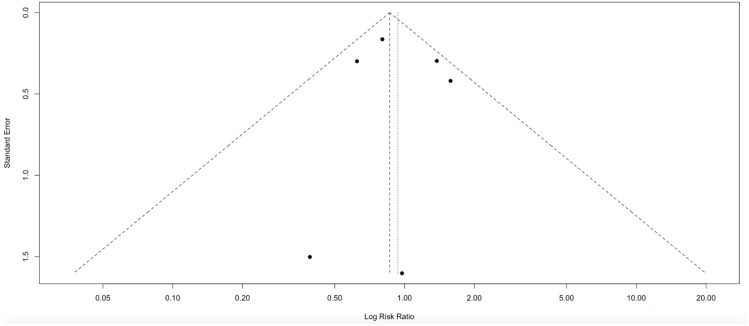
Funnel plot assessing publication bias for readmission or reoperation outcomes

PPFx risk was reported in 6 studies (*n* = 10,460). The pooled PPFx risk during the follow-up period was 0.9% (95% CI 0.5–1.5) for patients with EDS and 0.7% (95% CI 0.6–0.9) for non-EDS patients. Fixed-effect meta-analysis suggested a higher fracture risk in the EDS group, although this did not reach statistical significance (RR = 1.40 (95% CI 0.78–2.51), *p* = 0.266) (Fig. 6). In subgroup analyses, no statistically significant difference was observed in TKA studies (RR = 0.72, 95% CI 0.28–1.86, *p* = 0.502) whereas THA studies suggested a higher risk of PPFx risk in the EDS group (RR = 2.75, 95% CI 1.24–6.08, *p* = 0.012).

### Medical Complications

**Fig. 13:**
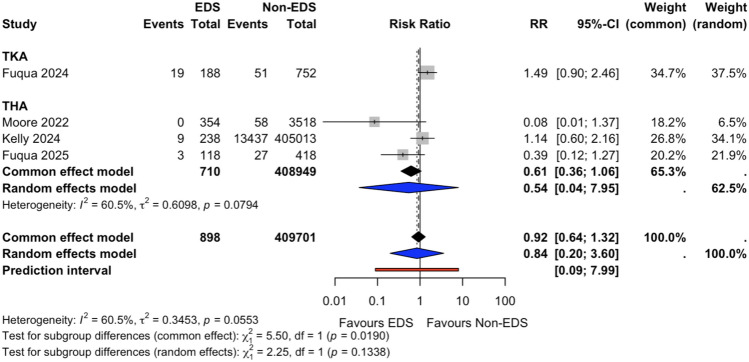
Forest plot comparing medical complication risk between patients

Medical complication risk was reported in 4 studies (*n* = 410,599). The pooled medical complication risk during the follow-up period was 3.5% (95% CI 2.4–4.9) for patients with EDS and 3.3% (95% CI 3.3–3.4) for non-EDS patients. Meta-analysis suggested no clear difference in medical complication risk between groups (RR 0.84, 95% CI 0.20–3.60, *p* = 0.733; I^2^ = 0.6%) (Fig 13). Fixed-effect sensitivity analysis yielded consistent findings (RR 0.92, 95% CI 0.64–1.32, *p* = 0.647). In subgroup analyses, no statistically significant difference was observed in TKA studies (RR 1.49, 95% CI 0.90–2.46, *p* = 0.119), or THA studies (RR 0.54, 95% CI 0.04–7.95, *p* = 0.430; I^2^ = 0.6%).

### Periprosthetic Joint Infection

**Fig. 14:**
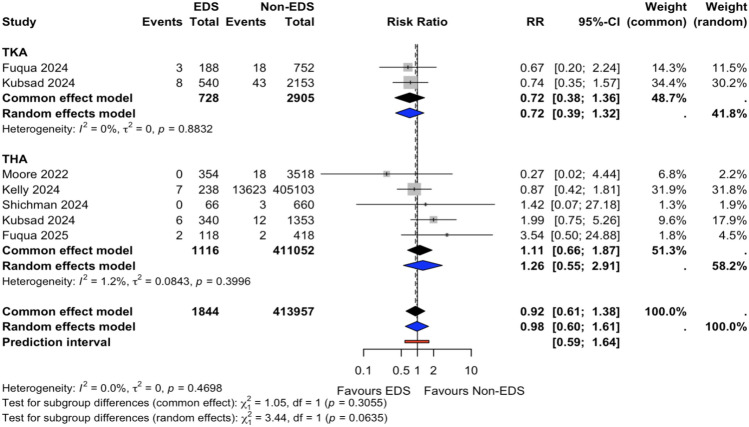
Forest plot comparing periprosthetic joint infection risk between patients

PJI risk was reported in seven studies (*n* = 415,801). The pooled PJI risk during the follow-up period was 1.4% (95% CI 0.9–2.1) for patients with EDS and 3.3% (95% CI 3.3–3.4) for non-EDS patients. However, the crude pooled non-EDS proportion is heavily influenced by one large database study (Kelly et al., 2024) contributing 97.9% of non-EDS patients. Meta-analysis suggested no clear difference in PJI risk between groups (RR 0.98, 95% CI 0.60–1.61, *p* = 0.919; I^2^ = 0.0%) (Fig. 14). Fixed-effect sensitivity analysis demonstrated similar results (RR 0.92, 95% CI 0.61–1.38, *p* = 0.681). In subgroup analyses, no statistically significant difference was observed in TKA studies (RR 0.72, 95% CI 0.39–1.32, *p* = 0.092; I^2^ = 0.0%), or THA studies (RR 1.26, 95% CI 0.55–2.91, *p* = 0.477; I^2^ = 0.0%). Fixed-effect subgroup sensitivity analyses were consistent.

### Readmission or Reoperation

**Fig. 15:**
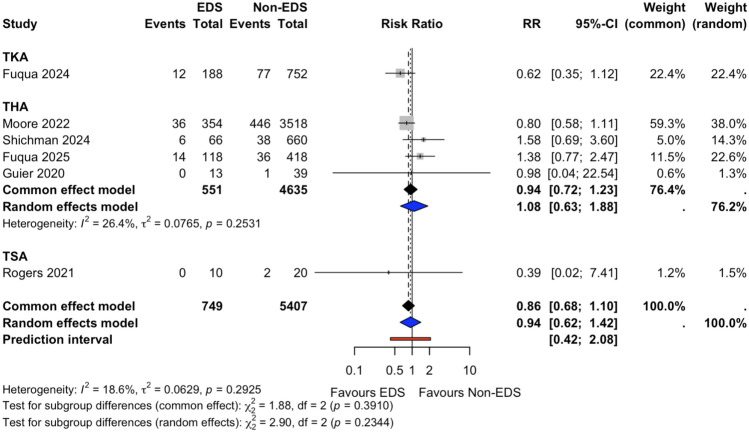
Forest plot comparing readmission or reoperation risk between patients

Readmission or reoperation risk was reported in six studies (*n* = 6156). The pooled readmission or reoperation risk during the follow-up period was 9.1% (95% CI 7.1–11.4) for patients with EDS and 11.1% (95% CI 10.3–12.0) for non-EDS patients. Meta-analysis suggested no clear difference in the risk of readmission or reoperation between groups (RR 0.94, 95% CI 0.62–1.42, *p* = 0.698; I^2^ = 0.2%) (Fig. 15). Fixed-effect sensitivity analysis yielded consistent findings (RR 0.86, 95% CI 0.68–1.10, *p* = 0.228). In subgroup analyses, no statistically significant difference was observed in TKA studies (RR 0.62, 95% CI 0.35–1.12, *p* = 0.115; I^2^ not applicable), or THA studies (RR 1.08, 95% CI 0.63–1.88, *p* = 0.674; I^2^ = 0.3%). Fixed-effect subgroup sensitivity analyses were consistent.

### Wound Complications

**Fig. 16:**
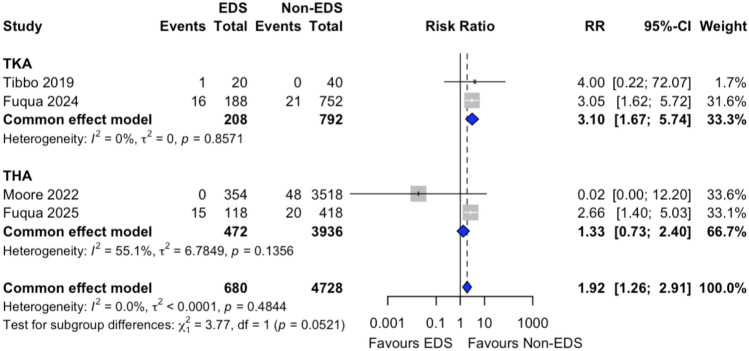
Forest plots comparing wound complication risk between patients

Wound complication risk was reported in four studies (*n* = 5408). The pooled wound complication risk during the follow-up period was 4.7% (95% CI 3.2–6.6) for patients with EDS and 1.9% (95% CI 1.5–2.3) for non-EDS patients. Fixed-effect meta-analysis suggested a higher risk of wound complications in the EDS group that reached statistical significance (RR 1.92, 95% CI 1.26–2.91, *p* = 0.002) (Fig. 16). In subgroup analyses, a statistically significant difference was observed in TKA studies (3.095, 95% CI 1.671–5.735, *p* = 0.000), but not THA studies (RR = 1.327, 95% CI 0.735–2.398, *p* = 0.348).

## Discussion

This systematic review and meta-analysis provides the most comprehensive synthesis to date of postoperative outcomes following primary total joint arthroplasty in patients with Ehlers–Danlos syndrome. The principal findings are that EDS is associated with a significantly increased risk of instability or dislocation and all-cause revision, while no clear differences were observed in periprosthetic joint infection, medical complications, readmission, or reoperation. Although higher absolute rates of aseptic loosening, periprosthetic fracture, and wound complications were observed in patients with EDS, these findings were not consistently statistically significant in the primary random-effects analyses and should be interpreted with caution.

The nearly threefold increased risk of instability or dislocation observed across pooled analyses is consistent with the underlying pathophysiology of EDS. Generalised ligamentous laxity, deficient capsuloligamentous support, and abnormal collagen architecture may compromise soft tissue restraint around prosthetic joints, predisposing patients to early and recurrent instability [23]. Importantly, this association was consistent across both THA and TKA subgroups and demonstrated minimal heterogeneity, suggesting a stable effect across joints, study designs, and analytic models. These results align with prior single-database and matched cohort studies reporting elevated dislocation rates in EDS and extend these observations across arthroplasty procedures using pooled comparative data [11, 14, 15].

Patients with EDS also demonstrated a significantly increased risk of all-cause revision, particularly following total hip arthroplasty. Revision is a multifactorial outcome, but the absence of increased infection or medical complication risk suggests that revision may be more closely related to mechanical complications, including instability and implant-related failure. While aseptic loosening and wound complications occurred more frequently in patients with EDS, these associations did not consistently reach statistical significance in the primary analyses, and may reflect limited statistical power, rare event rates, and variability in outcome definitions rather than definitive differences.

In contrast, no clear differences were observed in PJI or medical complications. Despite theoretical concerns regarding immune dysfunction, impaired healing, or multisystem involvement in EDS [10], the pooled data do not support an increased susceptibility to infection or systemic postoperative morbidity following arthroplasty. This finding is clinically important, as it suggests that EDS should not be viewed as a contraindication to arthroplasty when surgery is otherwise indicated, but rather as a condition that primarily modifies mechanical risk rather than systemic perioperative risk.

These results have direct implications for clinical practice. Preoperative counselling should explicitly address the elevated risk of instability and revision in patients with EDS, while reassuring patients that infection, medical complication, and readmission risks appear comparable to those of the general arthroplasty population. For THA, surgeons may consider strategies to reduce instability risk within the framework of shared decision-making: dual-mobility acetabular constructs and larger femoral head sizes have demonstrated reduced dislocation rates in other high-instability populations and may offer benefit in EDS; constrained liners represent an alternative where laxity is severe, albeit with their own trade-offs; and careful attention to component positioning, particularly acetabular cup anteversion and combined anteversion, alongside meticulous capsular repair, may further reduce instability risk. An anterior-based approach may also be considered given its association with lower dislocation rates in the general and high-risk populations, though direct EDS-specific evidence is limited. For TKA, surgeons should have a low threshold for using constrained implant designs—including posterior-stabilised, constrained condylar, or varus–valgus constrained implants—given the significantly elevated instability risk observed in this review; meticulous ligamentous balancing is particularly important. For both procedures, careful soft tissue handling and layered wound closure with attention to tension relief are advisable given the collagen fragility and impaired healing characteristic of EDS. Direct evidence for these strategies specifically in EDS patients remains limited, and recommendations should be considered in the context of individual clinical circumstances and patient preference.

This study has several limitations. All included studies were retrospective and relied largely on administrative or registry data, introducing potential residual confounding and misclassification bias. EDS was identified using diagnostic coding, precluding stratification by subtype or disease severity and raising the possibility of misclassification or dilution of true effect sizes. Follow-up durations were relatively short in most included studies, and pooled estimates for revision and aseptic loosening likely reflect early mechanical failures rather than long-term implant survivorship; late complications, including delayed aseptic loosening, fatigue fracture, and late instability, may therefore be underestimated.

For rare event outcomes (wound complications and periprosthetic fracture), fixed-effect Mantel–Haenszel models with treatment arm continuity correction (TACC) were used to provide more stable estimates; however, even with this approach, the sparse event data in several subgroups limits reliability, and future analyses incorporating larger datasets or Bayesian methods may yield more precise estimates. Definitions of instability and dislocation were not uniform across included studies; in THA, this primarily captured dislocation events via diagnostic codes, whereas in TKA studies broader clinical instability was encompassed, which may introduce heterogeneity in outcome ascertainment and limit cross-joint comparisons Additionally, outcomes beyond the hip and knee were sparsely reported, restricting joint-specific analyses.

## Conclusion

In summary, this systematic review and meta-analysis demonstrates that patients with Ehlers–Danlos syndrome undergoing total joint arthroplasty have significantly increased risks of instability or dislocation and all-cause revision compared with non-EDS patients. In contrast, no clear differences were observed in periprosthetic fracture, infection, medical complications, or readmission. These findings suggest that EDS primarily influences mechanical outcomes rather than systemic postoperative risk. Total joint arthroplasty remains an appropriate treatment option in patients with EDS, but enhanced preoperative counselling and strategies aimed at optimising joint stability may be warranted. Further prospective studies with longer follow-up and stratification by EDS subtype are needed to better define risks and guide management.

## Appendix

See Appendix Figs. 7, 8, 9, 10, 11, 12, 13, 14, 15 and 16

See appendix Table 2

**Table 2:** Summary of outcome reporting

Study	Joint	All-cause revision	Instability/dislocation	Aseptic loosening	Periprosthetic fracture	PJI	Medical complications	Readmission/reoperation	Wound complications	Outcomes reported
Guier 2020	THA	✓	✓	—	—	—	—	✓	—	3
Moore 2022	THA	—	✓	—	✓	✓	✓	✓	✓	6
Kelly 2024	THA	✓	✓	—	—	✓	✓	—	—	4
Shichman 2024	THA	✓	✓	✓	✓	✓	—	✓	—	6
Tibbo 2019	THA	✓	✓	✓	—	—	—	—	✓	4
Kubsad 2024	THA	✓	✓	✓	✓	✓	—	—	—	5
Kubsad 2024	TKA	✓	✓	✓	✓	✓	—	—	—	5
Fuqua 2024	TKA	✓	✓	✓	✓	✓	✓	✓	✓	8
Fuqua 2025	THA	✓	✓	✓	✓	✓	✓	✓	✓	8
Rogers 2021	TSA	✓	✓	—	—	—	—	✓	—	3
Studies per outcome	9	10	6	6	7	4	6	4		
